# Antenatal corticosteroids trial in preterm births to increase neonatal survival in developing countries: study protocol

**DOI:** 10.1186/1742-4755-9-22

**Published:** 2012-09-19

**Authors:** Fernando Althabe, José M Belizán, Agustina Mazzoni, Mabel Berrueta, Jay Hemingway-Foday, Marion Koso-Thomas, Elizabeth McClure, Elwyn Chomba, Ana Garces, Shivaprasad Goudar, Bhalchandra Kodkany, Sarah Saleem, Omrana Pasha, Archana Patel, Fabian Esamai, Waldemar A Carlo, Nancy F Krebs, Richard J Derman, Robert L Goldenberg, Patricia Hibberd, Edward A Liechty, Linda L Wright, Eduardo F Bergel, Alan H Jobe, Pierre Buekens

**Affiliations:** 1 Institute for Clinical Effectiveness and Health Policy (IECS), Dr. Emilio Ravignani 2024, Buenos Aires, C1414CPV, Argentina; 2 RTI International, 3040 Cornwallis Rd, Cox 229, Research Triangle Park, NC, 27709, USA; 3 Center for Research for Mothers and Children Eunice Kennedy Shriver, National Institute of Child Health and Human Development. National Institutes of Health, 6100 Executive Blvd, Room 4B09B, MSC 7510, Rockville, MD, 20852-7510, USA; 4 RTI International, 3040 Cornwallis Rd, Hill 320, Durham, NC, 27709, USA; 5 University Teaching Hospital, Lusaka, Zambia; 6 IMSALUD, 3ra calle A 6-56 zona 10, oficina 207, Guatemala City, 01011, Guatemala; 7 Department of Physiology, J N Medical College, Belgaum, Karnataka, 590 010, INDIA; 8 Department of Medical Education, J N Medical College, Belgaum, Karnataka, 590 010, INDIA; 9 KLEU Research Foundation, Jawaharlal Nehru Medical College, Belgaum, Karnataka, 590 010, INDIA; 10 Departments of Community Health Sciences, Aga Khan University Medical College, PO Box 3500, Stadium Road, Karachi, 74800, Pakistan; 11 Department of Pediatrics, Clinical Epidemiology Unit, Indira Gandhi Government Medical College, Opp Tidke Vidyalay, Katol Road, Nagpur, 440013, INDIA; 12 Moi University School of Medicine, PO Box 3900, Eldoret, 30100, Kenya; 13 Department of Pediatrics/Division of Neonatology, University of Alabama at Birmingham, 619 S 20th Street, 525 New Hillman, Birmingham, Alabama; 14 Pediatric Nutrition, University of Colorado Denver, Box C225, Research Complex II, 12700 East 19th Avenue, Rm 5026, Aurora, CO, 80045, USA; 15 Department of OB-GYN Christiana Care, 4755 Ogletown-Stanton Rd Room 1903, Newark, DE, 19718, USA; 16 Department of Obstetrics/Gynecology, Columbia University, 622 West 168th Street, PH16, New York, NY, 10032, USA; 17 Division of Global Health, Department of Pediatrics, Massachusetts General Hospital for Children, 50 Staniford Street, Suite 1054 125, Boston, MA, 02114, USA; 18 Department of Pediatrics, Indiana University School of Medicine, 699 West Drive, RR 208, Indianapolis, IN, 46202-5119, USA; 19 Center for Research of Mothers and Children, Eunice Kennedy Shriver National Institute of Child Health and Human Development, National Institutes of Health, 6100 Executive Blvd., Room 4B05J, MSC 7510, Rockville, MD, 20852-7510, USA; 20 Cincinnati Childrens Hospital, 3333 Burnet Ave, Cincinnati, OH, 45229, USA; 21 Tulane School of Public Health and Tropical Medicine, School of Public Health, 1440 Canal Street, Suite 2430, New Orleans, LA, 70112, USA

**Keywords:** Neonatal mortality, Antenatal corticosteroids, Implementation research, Preterm birth

## Abstract

**Background:**

Preterm birth is a major cause of neonatal mortality, responsible for 28% of neonatal deaths overall. The administration of antenatal corticosteroids to women at high risk of preterm birth is a powerful perinatal intervention to reduce neonatal mortality in resource rich environments. The effect of antenatal steroids to reduce mortality and morbidity among preterm infants in hospital settings in developed countries with high utilization is well established, yet they are not routinely used in developing countries. The impact of increasing antenatal steroid use in hospital or community settings with low utilization rates and high infant mortality among premature infants due to lack of specialized services has not been well researched. There is currently no clear evidence about the safety of antenatal corticosteroid use for community-level births.

**Methods:**

We hypothesize that a multi country, two-arm, parallel cluster randomized controlled trial to evaluate whether a multifaceted intervention to increase the use of antenatal corticosteroids, including components to improve the identification of pregnancies at high risk of preterm birth and providing and facilitating the appropriate use of steroids, will reduce neonatal mortality at 28 days of life in preterm newborns, compared with the standard delivery of care in selected populations of six countries. 102 clusters in Argentina, Guatemala, Kenya, India, Pakistan, and Zambia will be randomized, and around 60,000 women and newborns will be enrolled. Kits containing vials of dexamethasone, syringes, gloves, and instructions for administration will be distributed. Improving the identification of women at high risk of preterm birth will be done by (1) diffusing recommendations for antenatal corticosteroids use to health providers, (2) training health providers on identification of women at high risk of preterm birth, (3) providing reminders to health providers on the use of the kits, and (4) using a color-coded tape to measure uterine height to estimate gestational age in women with unknown gestational age. In both intervention and control clusters*,* health providers will be trained in essential newborn care for low birth weight babies. The primary outcome is neonatal mortality at 28 days of life in preterm infants.

**Trial registration:**

ClinicalTrials.gov. Identifier: NCT01084096

## Background

One of the United Nations Millennium Summit goals is a two-thirds reduction, by 2015, in death among children under 5 years old [1]. Given that 38% of all under-5 deaths worldwide occur in the first 4 weeks of life, the goal seems unattainable unless a significant fraction of the neonatal deaths are prevented [2]. Thus, the provision of perinatal health care in developing countries is a top priority. Preterm birth is a major cause of neonatal mortality, currently responsible for 28% of the deaths overall and is the second most common cause of death of children under 5 years old [2].

### Interventions to reduce neonatal mortality: antenatal corticosteroids

The most effective perinatal intervention to reduce neonatal mortality is the administration of antenatal corticosteroids to pregnant women at high risk of preterm birth [3]. A high-quality systematic review including 18 trials [4] found a 34% relative reduction in the incidence of Respiratory Distress Syndrome (RDS) (risk ratio [RR] 0.66, 95% confidence interval [CI] 0.59–0.73), a 46% relative reduction in intraventricular hemorrhage (RR 0.54, 95% CI 0.43–0.69), and a 31% relative reduction in neonatal mortality (RR 0.69, 95% CI 0.58–0.81). These benefits were confirmed irrespective of infant gender or race. The trials conducted in specific subpopulations in developing countries (preeclamptic women, or women with Premature Preterm Rupture of Membranes (PPROM)) in Brazil, Jordan, South Africa, and Tunisia, showed similar results with estimates of the effect within the same 95% CIs [5].

There is no evidence of short-term adverse effects of antenatal corticosteroids for the infants. In the subpopulation of PPROM infants, there is no evidence of an increased risk of infection (RR 1.05, 95% CI 0.66–1.68) [6]. There also has been no evidence of increased risk of serious adverse events in mothers who received corticosteroids [3]. The risk of maternal infection was a concern, specifically in women with PPROM, but a systematic review conducted by Harding et al. examined 15 trials with more than 1,400 women with PPROM and found no increased risk of infection (RR 0.86, 95% CI 0.61–1.20) [6]. Maternal pulmonary edema can occur when antenatal corticosteroids are used in combination with tocolytic agents. This complication is relatively rare and more commonly associated with maternal infection, fluid overload, and multiple gestations. Pulmonary edema has not been reported when antenatal corticosteroids are used alone.

To date, the trials of maternal corticosteroids have been conducted in hospital settings. There is a need to verify that the safety of corticosteroids would be similar for births occurring at home or in community health centers and where referral systems or advanced care for preterm births are limited.

#### Use in low-middle income countries

One indicator of the coverage of this intervention is the proportion of preterm babies whose mothers received the antenatal corticosteroids in a defined place and period. Even in high-income countries, a proportion of women at risk of preterm delivery do not receive antenatal corticosteroids because of lost opportunities or advanced labor, yielding an assumption of 80% maximum rates of the intervention for preterm deliveries below 34 weeks of gestational age (GA) [2,7,8]. In contrast, in 2000 in the 42 countries with 90% of the worldwide childhood deaths, only about 5% of mothers of preterm newborns received antenatal corticosteroids [9]. Another study based on data from 75 countries estimated that 10% of appropriate candidates received corticosteroids [2]. In Latin America, six studies conducted between 1999 and 2009 reported that the use of antenatal corticosteroids in preterm babies ranged between 4% and 71% [10-15].

#### Factors and causes of underuse

Why, despite the evidence of the past 30 years, are antenatal corticosteroids still not used routinely in low-middle income countries? There is a need for studies to address the contextual factors that affect the delivery of antenatal corticosteroids and the achievement of high and equitable coverage. The evidence supporting the use of antenatal corticosteroids comes from trials that were conducted in health facilities with neonatal intensive care units. Therefore, there is no clear evidence that the same benefits would be found in settings in which preterm babies could not receive appropriate care, such as primary health care centers or home births. It is likely that some of the following factors are associated with the lack of use: (1) Lack of availability of the drug; (2) Lack of guidelines and local regulations about who can administer injections (In the Global Network settings, Traditional Birth Attendants (TBAs), who are not allowed to administer injections, attend 40% of the deliveries); (3) Health providers’ lack of knowledge about of the beneficial effects of antenatal corticosteroids and misconceptions that corticosteroids produce adverse effects; (4) Difficulty identifying women at high risk of preterm birth in these settings; (5) Lack of accessibility to an appropriate level of care. At the hospital level in low-middle income countries, the major causes of underuse are the lack of availability of the drug and the lack of an active strategy to promote its use.

### Rationale for the trial

The effect of antenatal corticosteroids to reduce mortality and morbidity among preterm infants delivered in hospital settings in high-income countries is well-established [4]. However, the impact of increasing antenatal steroid use in hospital or community settings without access to specialized services has not been well researched. Similarly, the safety of corticosteroids is well proven in women delivering at the hospital level; however, there is currently no clear evidence about the safety of antenatal corticosteroid use for community-level births.

This is a pragmatic cluster, randomized controlled trial designed to evaluate the effects of a multifaceted intervention that will improve the identification of pregnancies at high risk of preterm birth and will provide and facilitate the appropriate use of corticosteroids to women at risk of preterm delivery, with the goal of reducing neonatal mortality rates among preterm infants in communities with low antenatal corticosteroid use. The intervention will target the health system as whole, not individual patients.

Our hypothesis is that the multifaceted intervention will improve the identification and referral of pregnant women at high risk of preterm birth, increase the availability of antenatal corticosteroids and facilitate their appropriate use in eligible women and thus reduce neonatal mortality at 28 days in preterm newborns by 25%. We also hypothesize that the therapy will be safe for women and their newborns. Dexamethasone phosphate is the drug of choice for this study because it is readily available in the participating countries. This drug is used as the standard of care at the hospital level for women at risk of preterm birth at many of the sites. The primary objective will be to evaluate whether a cluster-level multifaceted intervention, including components to improve the identification of pregnancies at high risk of preterm birth and to provide and facilitate the appropriate use of corticosteroids, reduces neonatal mortality at 28 days of life in preterm newborns, compared with the standard delivery of care. The secondary objective is to determine whether the intervention is safe for women and their newborns.

### Methods/design

**Figure 1:**
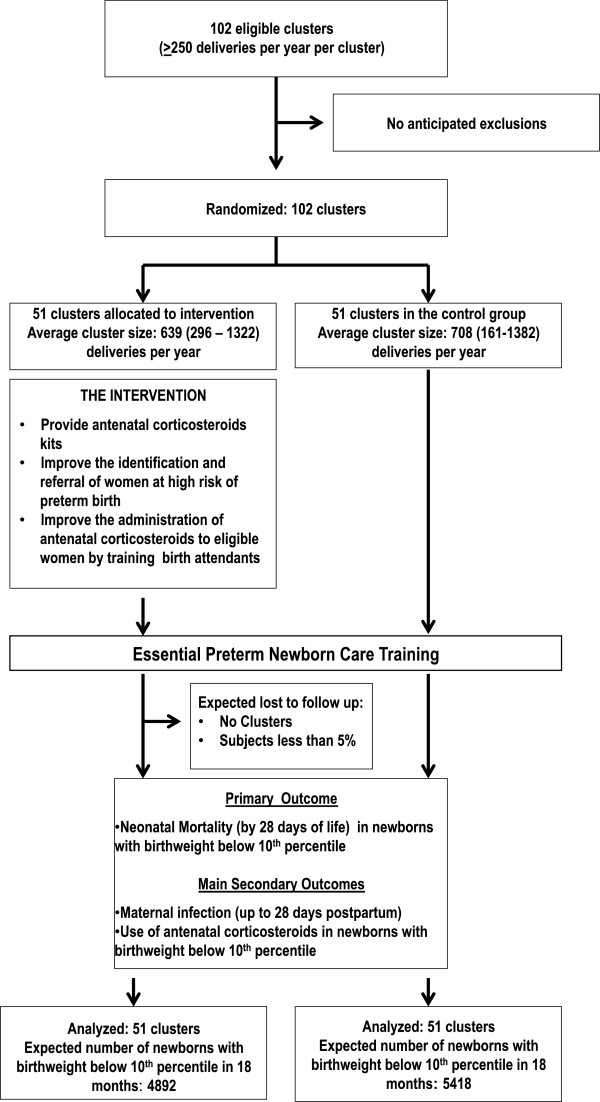
** Trial Design.** Design of the cluster randomized controlled trial to increase the use of antenatal corticosteroids to improve neonatal survival in developing countries.

The study design is a two-arm, parallel cluster, randomized controlled trial. Clusters will be randomized 1:1 to an intervention group or to a control group. Specifically, the intervention consists of the following: (1) training birth attendants to identify risk factors for preterm birth and to determine eligibility for antenatal corticosteroids (2) using a color-coded tape to measure uterine height to improve the assessment of gestational age (GA), and (3) distributing kits with vials of corticosteroids and unique reuse prevention syringes to all birth attendants for use in the community. Neonatal mortality at 28 days will be measured for all low birth weight (LBW) infants in control and intervention clusters for a period of 18 months. LBW will be used as a proxy for preterm birth because in Global Network sites approximately 15% of the women who delivered at health centers or homes did not know their last menstrual period LMP dates, and 40% of the deliveries occurred at the community level, where ultrasound is not available (Figure 1).

### Participating clusters

The Global Network for Women’s and Children’s Health Research (GN) is comprised of seven geographical study sites, located in low- and middle-income countries, which function as research units for the various maternal and neonatal studies implemented by the network. Each site has research clusters, with 6 clusters in Argentina, 10 clusters in Zambia, 10 clusters in Guatemala, 20 clusters in Belgaum, India, 20 clusters in Nagpur, India, 20 clusters in Pakistan and 16 clusters in Kenya. Seven GN sites will participate in the ACT study and 102 clusters will be available for the implementation of this protocol. The sizes of the clusters differ across and within the GN sites, but each cluster was selected because it had 300 to 500 deliveries per year per cluster. The GN clusters include both rural and urban settings, with deliveries taking place at home and in health facilities. At this time, 40% of deliveries in the participating clusters occur in home or clinic settings with TBAs.

This is an intent-to-treat design and thus all pregnancy outcomes of women who deliver in the study clusters and provide consent will be collected. Cluster-level inclusion criteria include GN clusters with at least 250 deliveries per year that consent to participate. Participant-level inclusion criteria include all pregnant women living in and delivering in the study cluster, and identified as having high risk for preterm birth who are eligible to receive intramuscular corticosteroids and, in settings where it is not the standard of care, provide consent.

### Randomization procedures

Data from the Maternal and Neonatal Health Registry (MNH) - a prospective, population-based study of pregnancy outcomes at the seven GN sites - will be used as a baseline to select the clusters and to ensure that the underlying neonatal mortality rates are reasonably balanced across arms through the randomization procedure [16]. The 102 clusters will be allocated to the control or intervention groups. Because the GN sites differ substantially, simple random allocation may not provide adequate balance in the groups [17]. To address this problem, we will implement a stratified randomization procedure with strata defined based on GN site, participation arm in a previous GN trial, the Emergency Obstetric and Neonatal Care trial [18], and neonatal mortality rate. The number and size of the clusters, as well as the balanced randomization procedure will make a clinically important imbalance in relevant prognostic factors unlikely.

### The intervention

#### General outline

The study intervention will be (1) to provide antenatal corticosteroids kits (ready-to-use boxes containing corticosteroid vials, syringes, gloves, and instructions for administration) (2) to improve the identification and referral of women at high risk of preterm birth by training birth attendants to identify eligible women, (3) to post eligibility reminders at health facilities and in the boxes, (4) to measure uterine height with a color-coded tape to estimate GA in women with unknown GA, (5) to improve the administration of antenatal corticosteroids to eligible women by training birth attendants on its administration and encouraging its use through reminders at all primary health care levels and at all health care levels in which women at high risk of preterm birth could be attended.

#### Provision of antenatal corticosteroid kits

**Figure 2:**
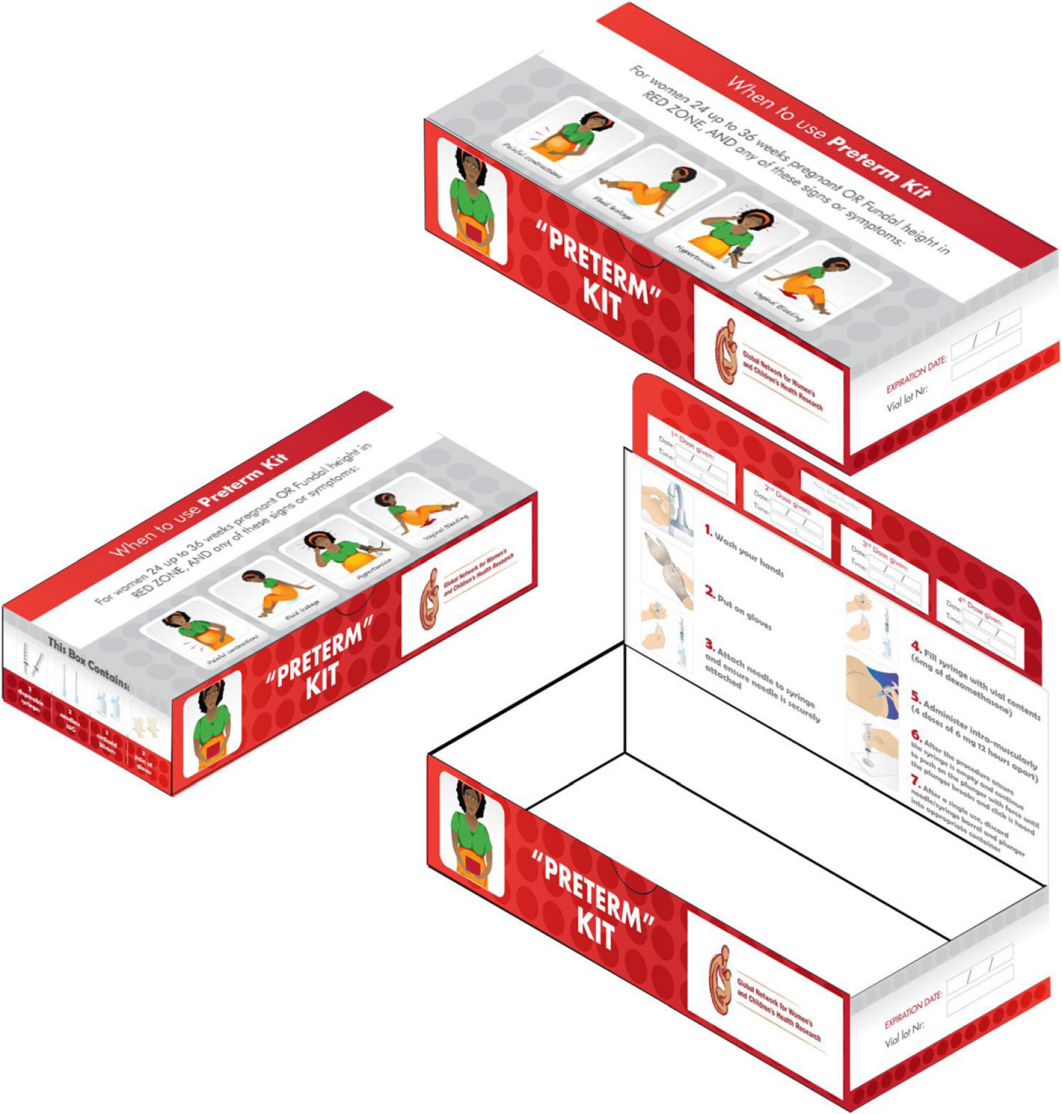
** Preterm Kit.** Ready-to-use treatment kits containing a full course of antenatal corticosteroids (4 vials with 6-mg of dexamethasone), 4 reuse prevention syringes, 4 pairs of gloves, and detailed instructions on when, how, and to whom to administer antenatal corticosteroids.

The study will distribute ready-to-use treatment kits containing a full course of antenatal corticosteroids (4 vials with 6-mg of dexamethasone phosphate), 4 reuse prevention syringes, 4 pairs of gloves, and detailed instructions on when, how, and to whom to administer antenatal corticosteroids (Figure 2).

The kits will be distributed in all health care facilities and delivery settings where eligible women might attend. Once an eligible pregnant woman is identified and has already given her consent, the birth attendant will administer the first dose of corticosteroids intramuscularly. The remaining 3 doses of dexamethasone will be administered at 12-hour intervals, if the delivery has not occurred, by either the same or another birth attendant. After administration of the first dose, the remaining vials will remain with the pregnant woman, along with a reminder for a health provider to administer the remaining doses, in case the woman is referred to another level of care or a different attendant is with her after 12 hours.

At the community level, the kits will be stored at the primary health center with the health providers or brought by the birth attendants when attending a home birth to ensure that eligible women have access to the medication. TBAs will be trained to identify pregnant women at high risk of preterm delivery and a communication strategy will be put in place so that a qualified health care provider is promptly informed of the situation.

All pregnant women who are between 24 weeks and 36 weeks GA will be eligible to receive antenatal corticosteroids if they present with any of the following: signs of labor, or threatened labor, amniotic fluid leakage, hemorrhage, or hypertension/severe headache.

### Reminders to health care providers

**Figure 3:**
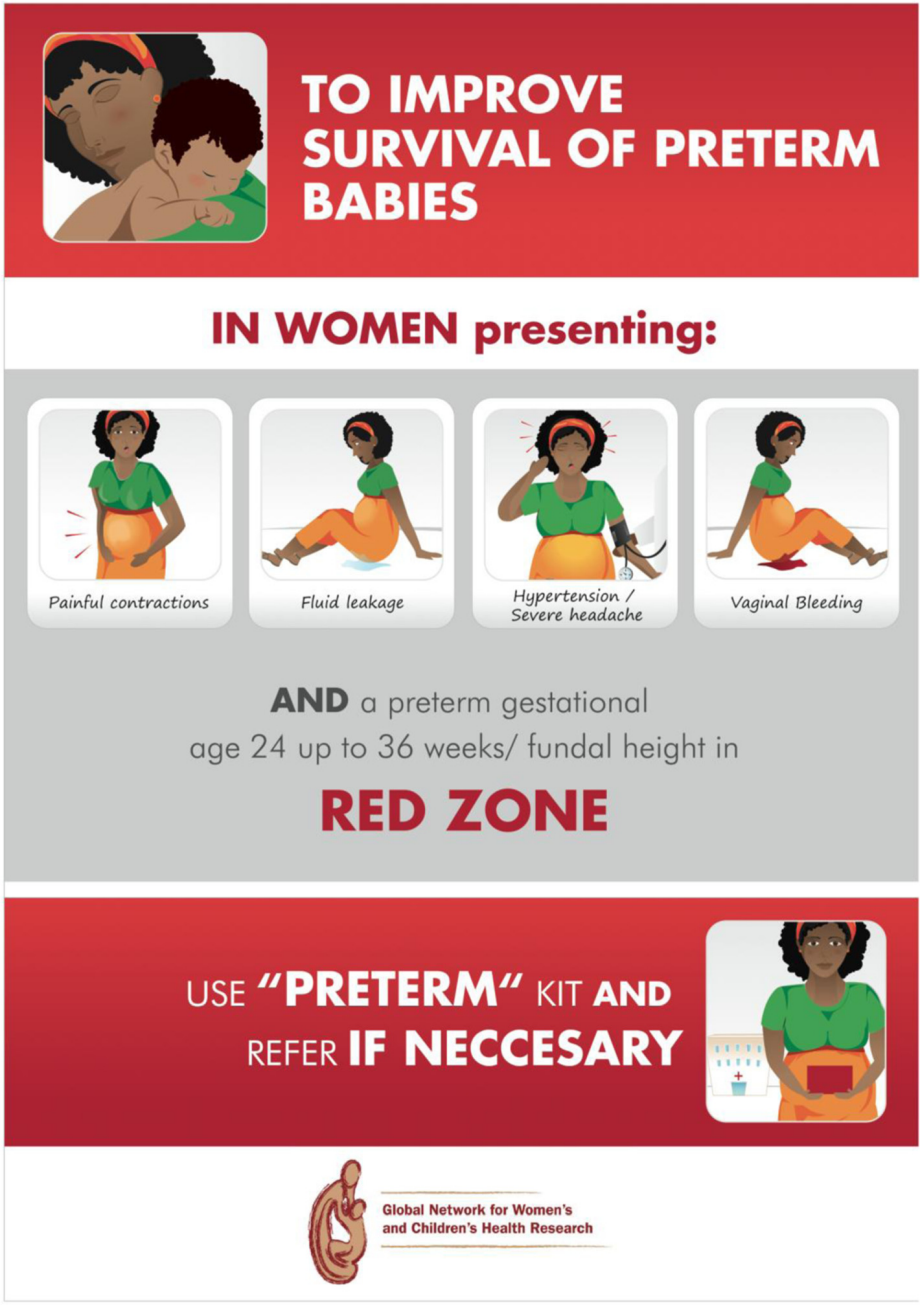
** Poster Reminder.** Poster to be placed in areas of care, including recommendations emphasizing the importance of providing antenatal corticosteroids to eligible women and why and whom to treat.

Reminders in poster format will be placed in areas of care (Figure 3). They include recommendations emphasizing the importance of providing antenatal corticosteroids to eligible women and why and whom to treat. Contents and design were developed during the preparatory phase, including translation to local languages and tailoring to the characteristics of the different participating sites.

### Improving the identification of eligible women

Treatment (antenatal corticosteroid injection) and referral will be recommended for all pregnant women who are between 24 and 36 weeks GA and present with any of the following [19]: *Signs of labor, or threatened labor* (intermittent abdominal pain, pain associated with blood-stained mucus discharge, watery vaginal discharge or a sudden gush of water, or any combination of these), *amniotic fluid leakage* (watery vaginal discharge or a sudden gush of water), *hemorrhage* (vaginal bleeding with or without cramping), *hypertension* (diastolic blood pressure of 90 mmHg or more on two consecutive readings taken at least 4 hours apart or 110 mmHg or more on one reading). In communities where blood pressure cannot be measured, women with eclampsia or signs or imminent eclampsia (e.g. severe headache) will be offered corticosteroids.

In some of the study communities, it is expected that an estimation of GA in women with signs of labor or pregnancy complications will not be available. Preliminary data from a previous GN study suggest that misclassification of preterm deliveries is significant [20]. Additionally, data from the GN sites suggest that approximately 15% of women giving birth at the community level do not know the date of their LMP.

**Figure 4:**
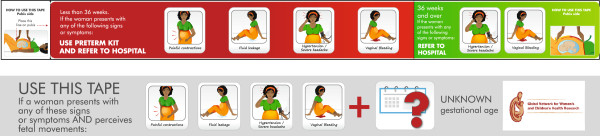
** Color-codedz tape.** Both sides of the tape to measure uterine height and estimate gestational age in women with unknown gestational age. Women with uterine height on the red zone are eligible to receive antenatal corticosteroids.

**Figure 5:**
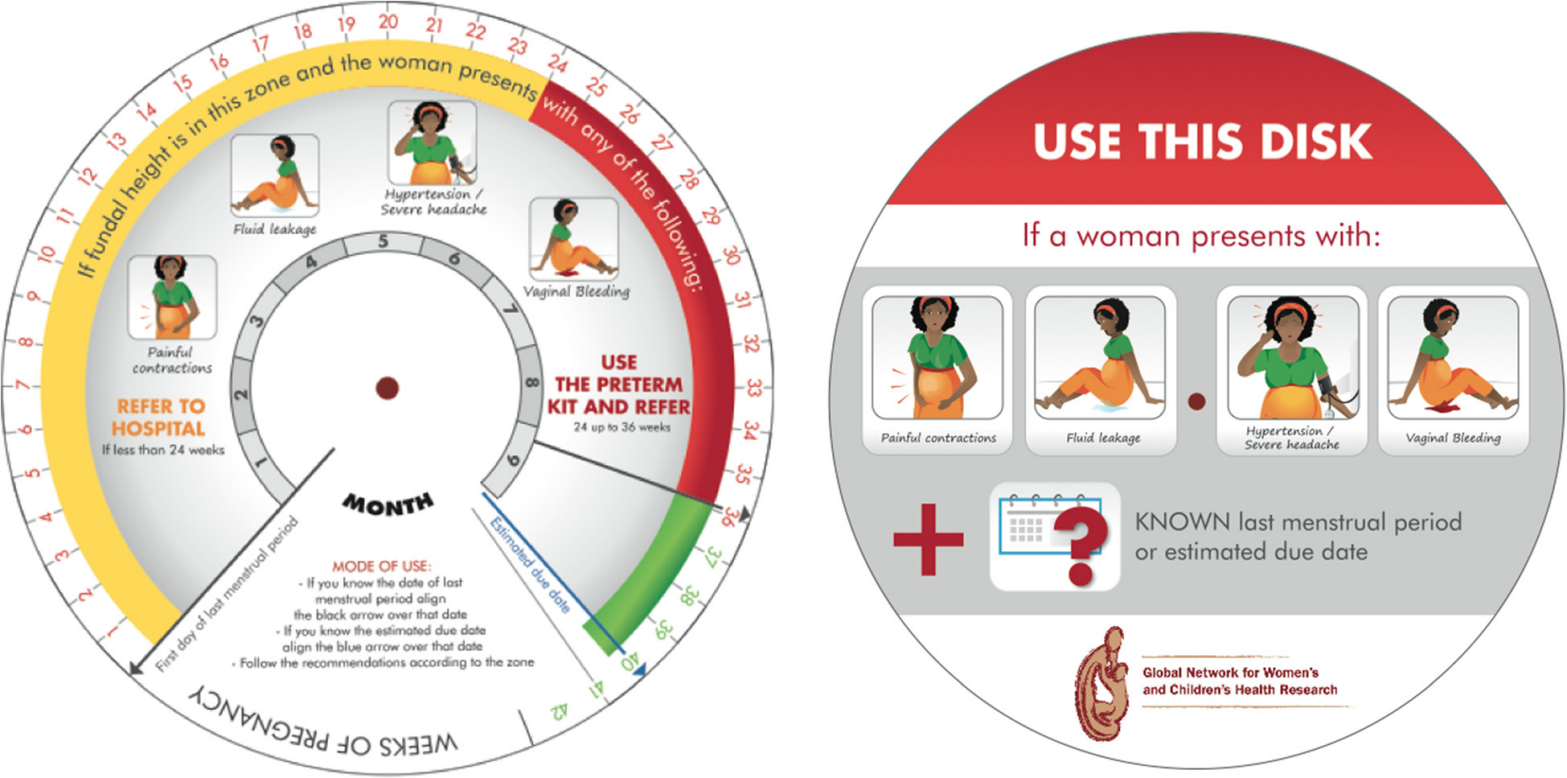
** Obstetric Disk.** Disk to assess gestational age and facilitate the detection of eligible women. Women with gestational age in the red zone are eligible to receive antenatal corticosteroids.

As part of the development of the intervention, we conducted a study to test a color-coded tape that we developed to identify preterm pregnancies between 24 and 36 weeks GA through fundal height measurement. Uterine fundal height measured by the color-coded tape will be used as a proxy for GA when reliable GA assessment is not available (Figure 4). The tape can be used in all pregnant women presenting with any of the risk factors and an unknown GA. The tape is divided in two segments, each shaded a different color; one segment clearly indicates the uterine height corresponding to the 24–36 weeks GA range. If LMP or estimated date of delivery is known, GA will be assessed using a specially designed obstetric disk (Figure 5). The use of the tape and obstetric disk will not be promoted for women with an ultrasound report verifying GA.

### Intervention training

All the health providers potentially involved in delivery attendance at intervention clusters will be trained to deliver the intervention during an interactive *train-the-trainer workshop.* Workshop participants will learn how to (1) identify those women eligible for antenatal corticosteroids administration and (2) appropriately use the preterm kit, if legally allowed to administer injections, and (3) refer the woman to a health center or contact a skilled birth attendant when indicated. We will adapt the workshops’ format according to the targeted health providers.

All birth attendants working in the intervention clusters are eligible for participation in the trial if they provide care to pregnant women, assist in the delivery of babies, and assist the women in caring for their babies. We anticipate that approximately 3,000 birth attendants will be trained. All eligible birth attendants will be fully briefed on the study before training and will be asked to sign a Decline Form if they are not willing to receive the training.

### Post-randomization components

Both the intervention and control communities will receive training in Essential Newborn Care for LBW Babies [20]. The purpose of this component is to ensure that all health care providers at study sites that attend the mother or baby are trained to use or promote the use of a minimum set of evidence- based interventions for the care of LBW and preterm infants born at homes or health care centers. The selected interventions are (1) neonatal resuscitation, (2) thermal regulation through skin-to-skin contact with the mother, (3) breastfeeding, and (4) referral if needed.

### Outcome measures

The primary outcome measure is neonatal mortality at 28 days of life in preterm infants in all clusters. LBW will be used as a proxy for preterm birth because in GN sites approximately 15% of the women who delivered at health centers or homes did not know their LMP dates, and 40% of the deliveries occurred at the community level, where ultrasound is not available. Thus, pragmatically, it is not possible to use gestational age to classify preterm births in these settings.

The variable to be used for classifying preterm births needs to be objective and unbiased in both intervention and control clusters. Thus it should be unrelated to the experimental intervention. The color-coded tape (Figure 4) is part of the intervention and will be used to improve the identification of pregnancies at high risk of preterm birth, but only in the intervention group. Therefore it is expected that this group will show more reliable gestational age assessment during the intervention period. Consequently, using gestational age to select the preterm group will likely introduce selection bias in the analysis.

Birth weight can be measured equally in both intervention and control clusters, and it is unrelated to the intervention. Birth weight measurement in the GN clusters is currently being done with high coverage (90%) and good quality. It is likely that some misclassification with small-for-gestational age (SGA) babies will exist, and that this misclassification can underestimate the real benefit of corticosteroids. But it is a conservative approach that will not introduce bias to the comparison between groups.

GN sites have different mean birth weights; those from Asian sites are much lower than those from African and Latin American sites. Using the same birth weight (e.g., < 2500 g) as a cutoff point at all sites would include a higher proportion of Asian babies, whose mean birth weights are ~2700 g, and most of them would be SGA babies rather than preterm. To avoid this, we will use different cutoff points for each country to select the 10% smallest babies in each GN site. Using data from the MNH study for each GN site, we set a birth weight cutoff point, rounded to 100 g, that would select the smallest 10% of infants as a proxy for preterm births (< 36 weeks GA). Using this rule, the cutoff point for sites in Argentina, Zambia, and Kenya was 2500 g; for sites in Guatemala and Karnataka, India, was 2400 g; and for sites in Nagpur, India, and Pakistan was 2200 g. The overall prevalence of LBW for all sites according to those weight limits is 8.2%, with an 11.4% neonatal mortality at 7 days. A birth weight of 750 g will be used as a cutoff point for the lower birth weight limit for analysis purposes, except for those GN sites where babies with birth weights < 1000 g are considered abortions.

The primary and secondary outcomes in LBW babies, the outcomes in all infants and their mothers, and the process outcomes (to be measured only in the intervention clusters) are shown in Table 1. LBW babies are defined as newborns with birthweight below the 10^th^ percentile of the site.

**Table 1:** Study outcomes

**Outcome measures in infants with birth weight below 10**^**th**^**percentile and their mothers**	**PRIMARY**
	- Neonatal mortality at 28 days
	**SECONDARY**
	- Rate of antenatal corticosteroid use
	- Maternal infection from birth up to 7 and 42 days postpartum
	- Perinatal mortality rate (stillbirths ≥ 20 weeks GA or ≥ 500 g + neonatal deaths before 7 days)
	- Early neonatal mortality rate at 7 days after birth
	- Mean neonatal weight at 7 and 28 days
	- Neonatal and perinatal mortality rates by country
	- Neonatal and perinatal mortality rates by type of setting (health facility based deliveries vs. community based deliveries)
	- Infant mortality rate at 42 days after birth*.*
**Outcome measures in all infants and their mothers**	- Early neonatal mortality (7 days after birth)
	- Neonatal mortality at 28 days after birth
	- Maternal infection from birth up to 7 days postpartum
	- Maternal infection from birth up to 42 days postpartum
	- Infant mortality rate at 42 days after birth
**Process measures**	- Number of women receiving corticosteroids and number of doses
	- Number of referrals
	- Number of health providers trained
	- Number of kits distributed
	- Health providers’ opinions about the kits
	- Number of kits fully used (all doses administered) at site
	- Number of kits partially used (1–3 doses of dexamethasone) at site

### Data collection and management

Outcome data for the ACT trial will be collected through the existing MNH registry in the GN sites. This registry includes all the outcome variables including data to 42 days postpartum. Forms specific for ACT were developed to collect process outcomes.

A weekly data transmission from the research units to the Data Center will be completed using the system and transmission procedures currently in place for GN studies at each site. The data entry software will be programmed to conduct range checks and simple consistency checks of the data. Double data entry verification will be used to check for ID and data errors. An error rate of less than 0.3% is considered acceptable.

The Data Management System is password protected. It will require a user ID and password to be entered to ensure that only trained, authorized personnel have access to the data.

### Data analysis

The primary analysis will compare post-intervention outcomes between intervention and control clusters, controlling for country, using two complementary approaches. First, a cluster-based permutation test that is appropriate for the stratified randomization process be used to develop a formal hypothesis test and interval estimates of the treatment difference between the two arms. A model-based analysis will be used to assess whether the inferences generated by the permutation test are robust enough to control for potential confounders and to describe any evidence of differential treatment effects in the different countries. Although this study is not powered to evaluate treatment by country interactions formally, any evidence of such interactions will be described through the model-based analysis of this primary outcome.

An intent-to-treat analytic approach will be used for both the permutation test and for the model-based analyses, in that any cluster assigned to the intervention will be considered treated, whether or not the ACT intervention was delivered in that cluster. Also, both analyses will use country as a blocking or stratification variable in the analysis to reflect the randomization scheme.

To facilitate the model-based primary analysis, a detailed analysis of baseline variables of the two groups (intervention and control clusters) will be performed to assess the extent, if any, of baseline imbalances between the two groups of clusters. If baseline differences between the treatment arms are identified, the covariables representing the imbalanced variables will be used for adjusted analyses. Imbalances at the cluster level (e.g., proportion of hospital births, type of birth attendant) can be adjusted in the model-based analysis. Because the primary interest is in the decrease in the marginal neonatal mortality rate at the cluster level, the generalized estimating equations (GEE) method of Liang and Zeger [21] as implemented through the SAS procedure GENMOD will be used for the primary analysis. The model will control for cluster-level effects by assuming country-specific correlation within clusters, and all inferences will be based on the empirical correlation matrix proposed by Liang and Zeger [21]. Additional details of the model will be specified in the Statistical Analysis Plan.

In addition to the permutation tests and GEE models described above, which focus on marginal risk in the population, we will evaluate the effect of the outcome on subject level risk by modeling the binary outcome using generalized linear mixed models as implemented through the SAS procedure GLIMMIX. In this procedure, cluster will be treated as a random effect, with the cluster-specific risk nested within intervention assumed to have a Gaussian distribution. Again, both the GEE model and the generalized linear mixed model will examine the effects of potential confounders on mortality risk and the heterogeneity of treatment effects across country.

#### *Secondary analyses—antenatal corticosteroid use*

Although the primary study outcome is neonatal mortality, a key secondary outcome is rate of antenatal corticosteroid use. The analytic approach for this outcome will be comparable with that described for the primary outcome in that both permutation and model-based analyses will be implemented.

The primary results likely will need to be substantiated with a sensitivity analysis of the results to missing outcome data. The need for a detailed sensitivity analysis will depend on the amount of missing data and any intervention-dependent pattern to the missing data. A first step will be to look for patterns by comparing observed characteristics of participants who have missing data with those having complete data. Then, if appropriate, both nonparametric and parametric approaches will be undertaken to evaluate the impact of missing data. Additional details will be provided in the Statistical Analysis Plan.

#### *Other secondary outcome measures*

The trial will also examine a number of additional clinical and process measures as secondary outcomes. For each of these outcome measures, descriptive statistics will be computed as described in the introductory paragraphs above. Model-based analyses will then be used to examine the effects of the intervention on each of clinical and process measures. For the clinical measures, GEE models will be used to evaluate the effect of the intervention on marginal risk of the outcome, with results reported for both unadjusted analyses and analyses controlling for covariates that differ across clusters. Also, generalized linear mixed models will be used to examine the effect of the intervention on subject-level risks. Again, results will be reported for both unadjusted and adjusted models.

### Sample size

#### *Effect size*

We expect to increase corticosteroids use from less than 10% to at least 50% among preterm births. From the estimates in the systematic review on the effect of corticosteroids on mortality [4], we expect an associated relative rate reduction in neonatal mortality of 25%–30%. The estimated effect size is lower than the effect observed in the systematic review of clinical trials [4], to take into consideration that we will use birth weight as a proxy for GA and therefore will include a proportion of term small for GA babies who may not benefit from receiving corticosteroids.

#### *Intracluster Correlation Coefficient (ICC)*

Using the MNH registry baseline data, the ICC is estimated to be between 0.01 and 0.05. The estimate was computed using data from 106 MNH registry clusters.

#### *Power estimates*

This trial will be conducted using all of the clusters from the MNH registry determined to have the local infrastructure to implement the protocol. On the basis of preliminary information from the site investigators, we anticipate between 90 and 100 clusters with at least 250 annual deliveries (average of 400) to be available for the trial. Assuming that between 8% and 9% of deliveries are premature, we anticipate a mean cluster size of between 50 and 75 premature newborns for an 18- to 24-month trial. For 28-day neonatal mortality, a study size of 90 clusters with at least 60 LBW deliveries per cluster will have a power of slightly more than 80% (Type I error rate of 0.05) to detect a decrease of 30% in neonatal mortality from 13% to 9%. For the outcome of antenatal corticosteroid use among LBW infants, a sample size of 90 clusters with 50 events per cluster has a power of greater than 0.99 to see an increase in utilization from a start-rate of 10%–20% or more.

### Ethical aspects

The use of antenatal corticosteroids for women at risk of preterm birth is the standard of care in high-income countries as well as at the hospital level for most of the countries participating in the trial. However, at each participating site, the Institutional Review Board (IRB), Ministry of Health, and physicians will be consulted on guidelines for the use of antenatal corticosteroids to ensure that we are in compliance with cultural, medical, and regulatory policies and norms. These drugs do not require regulatory approval in the participating countries, nor do they require approval from the Food and Drug Administration as an investigational new drug because the research is being conducted outside the United States.

The protocol and the informed consent documents were submitted and approved by the IRBs and ethics committees of all participating clinical sites and Research Triangle Institute (RTI), as the data center.

All participating communities must agree in advance to participate in the study. Community-responsible health authorities will provide their agreement to participate before beginning any activities and will act as cluster ethical guardians. Community education, using forums such as meetings and posters, will inform the community and its authorities about the purpose and procedures of the study. Communities will be informed of their assignment to the control or intervention cluster after randomization.

At the individual level, informed consent to collect data and to administer corticosteroids will be requested. Because high risk for preterm birth is frequently an emergency condition (preterm labor, hemorrhage), to the extent possible, informed consent to administer corticosteroids will be done during pregnancy for all women enrolled in the MNH registry. For those women eligible for corticosteroids administration who were not asked for consent during pregnancy, consent would be sought with identification of the risk of preterm delivery. In this case, women who present at the community or health center level with signs or symptoms that make them eligible to receive corticosteroids, will be consented only if they are in early labor (<6 cm of cervical dilation or the local equivalent), or in a clinical condition not evaluated as an extreme emergency (like severe hemorrhage, or imminent eclampsia).

At the hospital-level, where antenatal corticosteroids are the standard of care for women having high risk of preterm birth, eligible women will not require consent.

All health providers working in the intervention clusters that are eligible to be trained in the intervention procedures will be invited to receive the training and asked for their signed informed consent.

## Competing interests

The author(s) declare that they have no have competing interests.

## Authors' contributions

P Buekens, J Belizán, F Althabe and E Bergel had the original idea and designed the intervention and the first protocol. F Althabe, P Buekens, J Belizán, A Mazzoni, M Berrueta, J Hemingway-Foday, M Koso-Thomas and E McClure wrote the manuscript, in collaboration with E Chomba, A Garces, S Goudar, B Kodkany, S Saleem, O Pasha, A Patel, F Esamai, W Carlo, N Krebs, R Derman, R Goldenberg, P Hibberd, E Liechty, L Wright, and A Jobe. All authors have given final approval of the manuscript.
